# The One Health Epidemiology of Avian Influenza Infection in Bangladesh: Lessons Learned from the Past 15 Years

**DOI:** 10.1155/2023/6981327

**Published:** 2023-11-30

**Authors:** Mohammad Mahmudul Hassan, Pronesh Dutta, Md Mazharul Islam, Md. Ahaduzzaman, Shovon Chakma, Ariful Islam, Ricardo J. Soares Magalhaes

**Affiliations:** ^1^Faculty of Veterinary Medicine, Chattogram Veterinary and Animal Sciences University, Chattogram 4225, Bangladesh; ^2^Queensland Alliance for One Health Sciences, School of Veterinary Science, The University of Queensland, Gatton, QLD 4343, Australia; ^3^Department of Animal Resources, Ministry of Municipality, Doha, Qatar; ^4^EcoHealth Alliance, NY 10018, New York, USA; ^5^Institute of Epidemiology, Disease Control and Research (IEDCR), Dhaka 1212, Bangladesh

## Abstract

Avian influenza viruses (AIVs) are significant transboundary zoonotic pathogens that concern both animal and human. Since the first report of H5N1 AIV in Bangladesh in early 2007, it resulted in numerous outbreaks across the country, hindering the sustainable growth of the poultry industry through economic losses in different production systems (commercial and backyard). Highly pathogenic avian influenza (HPAI) virus and low pathogenic avian influenza (LPAI) virus are currently cocirculating and causing infection in poultry sectors in an endemic manner in Bangladesh as well as in wild bird species. The introduction of multiple clades of H5N1 in different poultry species and the reassortment of AIVs with different patterns of infections have complicated the epidemiological situation for control and created conditions to increase the virulence of the virus, host range, and potential zoonotic transmission. The risk of viral transmission at the human–poultry interface is increasing over time due to inadequate surveillance and early detection strategies and practices, ineffective biosecurity practices among poultry raisers, and the complex supply chains of backyard and commercial poultry and live bird market (LBM) systems. Improving AIV surveillance in poultry flocks and LBMs, vaccination, biosecurity, and awareness among poultry professionals is beneficial to controlling the disease burden in the poultry sector. However, human cases of AIV related to poultry production and marketing chain in Bangladesh suggest a One Health approach engaging various stakeholders from the public and private would be a better option for successfully controlling avian influenza outbreaks in Bangladesh. This review of literature presents the comprehensive overview of AIV infection status in Bangladesh, including a description of pathways for zoonotic transmission at different epidemiological interfaces, the genetic evolution of the virus, and the need for improvement of disease control strategies incorporated with early detection, application of effective vaccines, increases the proper biosecurity practices and improvement of awareness among the poultry raisers, traders and consumers using a One Health approach.

## 1. Introduction

Avian influenza virus (AIV) is a significant public health concern globally [1] besides in Bangladesh, infecting a wide range of avian species and causing a massive economic loss in the poultry industry, as well as human illness and death [2]. Bangladesh is one of the most impacted countries in the world concerning highly pathogenic avian influenza virus (HPAIV) outbreaks, mainly A/H5N1, in avian species [3]. The economic losses associated with the first reported HPAIV A/H5N1 outbreak in 2007 in Bangladesh have been estimated at US$746 million [4]. However, the low pathogenic avian influenza virus (LPAIV) A/H9N2 infection in poultry is common nowadays in Bangladesh, where mitigation is often complicated due to limitations in confirmatory diagnosis [5]. Since the first identification of LPAIV A/H9N2 infection in poultry in September 2006 [6], it has been detected frequently in multiple poultry species in Bangladesh [7]. Although LPAIV subtype cocirculate with the HPAIV A/H5N1 in Bangladesh [8], where the reassortment event between the HPAIV A/H5N1 and LPAI viruses is relatively uncommon [9]. However, LPAIV A/H9N2 has coexisted with HPAIV A/H5N1 in poultry throughout Bangladesh [10]. The human cases of both HPAIV H5N1 and LPAIV H9N2 were also very closely clustered genetically with chicken isolates, which indicates human infections are transmitted from chickens [11]. With the initiation of poultry surveillance in live bird markets (LBMs) and backyard poultry by different government and nongovernment organizations (NGOs) throughout Bangladesh, the detection of the AIV outbreak has been reported early and limited the spread of infection [12]. Since 2012, AIV outbreaks in poultry have declined dramatically, while only two outbreaks of H5N1 have been reported in 2013–2019 [13, 14]. Vaccination program against H5 was introduced in commercial poultry in 2012 primarily in two districts: Gazipur and Kishoreganj by the government authority and breeder associations [12]. However, a few vaccinated birds have been detected with shedding of virus through dropping [15, 16]. Vaccination failure may be observed due to inappropriate administration by incorrect dose, route, and proper cool chain maintenance. Besides, some other factors related to the virus, such as viral mutation, frequent changes of antigenic drift due to impaired immunity in vaccinated poultry, and immune escape are also responsible for vaccination failure [17–19]. Besides practicing strict biosecurity in commercial farms, the complex poultry production and marketing systems where poultry species share the same area may be the possible causes of becoming endemic of HPAIV H5N1 in Bangladesh [12, 20]. The complex communication between poultry production, and marketing, limited veterinary capacity, and the unwillingness of the poultry raisers to report suspected AIV cases in poultry to the government favor the persistence of AIVs, particularly HPAIV A/H5N1, in Bangladesh [21].

In Bangladesh, response protocols toward HPAIV A/H5N1 outbreaks in poultry in 2007–2012 were intended to stop and eliminate a newly introduced pathogen such as case detection followed by identification of premises deemed to be in direct contact with premises reporting infection, and subsequent stamping out of flocks with reported infection [17]. The response protocols of HPAIV A/H5N1 2007–2012 HPAIV were supported by a poultry farm-level active surveillance system comprised of teams of community animal health workers across the country tasked with monitoring poultry having HPAIV A/H5N1 clinical signs and reporting farm disease events to the livestock department [22]. Since then, the government and several NGOs have made multiple efforts to detect, track, and combat HPAIVs in the community [23]. The government policy of compensation initiated for the farmers whose birds were H5N1 identified and culled during the early outbreaks of AIV in Bangladesh [24], however, this compensation policy has now completely stopped due to inadequate funds [25, 26].

Among the poultry species, chickens are more susceptible to HPAIV A/H5N1, which causes high morbidity and mortality [27], while wild aquatic birds and domestic ducks are considered the natural reservoir of the virus [28–30], carrying and shedding the virus asymptomatically [31, 32]. Geographically, most outbreaks in the central part of Bangladesh are dominated by the commercial poultry production system, whereas most of the outbreaks in the backyard poultry system are reported from the northwestern part of Bangladesh [33]. Most HPAIV A/H5N1 outbreaks in Bangladesh have been reported in December, January, and February (winter season) [34]. Although Bangladesh has a large-scale trading system for poultry and poultry products, mixing the commercial and backyard poultry in LBMs for sale, where LBMs have reported with higher prevalence of AIV [35, 36]. Overcrowding and a constant supply of susceptible birds of various species and breeds may have promoted AIV silent transmission within the LBMs [37]. Moreover, the movements of wild and migratory birds influence the spread of AIVs in Bangladesh [38, 39]. Department of Livestock Services (DLS) has established the biosecurity guideline for poultry farms in Bangladesh [40], but poultry farmers are reluctant to follow recommended biosecurity guidelines, such as they have reported giving free access to wild birds and rodents, allowing the buyers, and vaccinators without applying disinfection, sharing the farms' equipment such as feeders, drinkers and weighing tools, disposal of dead and sick birds in open places, allowing the vehicles that carried chicks and feed to the farm without disinfection [17, 41, 42]. Besides, poultry raisers in commercial and backyard settings frequently come into close contact with sick poultry while handling and slaughtering without following any biosafety protocols [43]. Sometimes, poultry raisers sell or consume sick birds in the backyard poultry rearing system [43]. These types of practices promote the risk of AIV transmission at the human–bird interface in Bangladesh.

While much research has been conducted to reveal the epidemiology of HPAIV A/H5N1 in Bangladesh, it is vital to understand the state-of-play of cross-sector AIV epidemiology in Bangladesh since its introduction 15 years ago in early 2007. Hence, this narrative review is aimed to compile and critically appraise the current epidemiological evidence on AIVs in Bangladesh from a One Health perspective with a focus on (a) the dynamics of AIV transmission in sections of the poultry value chain and wetland ecosystems, (b) human infection patterns and determinants, and (c) the implementation and efficacy of intervention strategies to reduce AIVs transmission in Bangladesh.

## 2. Methods

A systemic search strategy was followed to identify all published articles related to narrative reports of the AIV in Bangladesh. Published articles were searched in five online electronic databases: PubMed, Web of Science, Embase, ProQuest, and Scopus, published between January 1972 and December 2022. The search was done using the following keywords: (Avian Influenza OR Bird Flu OR HPAI OR LPAI OR H5 OR H9 OR H5N1 OR H9N2) AND (LBM OR Live bird market OR Backyard poultry OR Household poultry OR Commercial poultry OR Broiler OR Layer OR Wild bird OR Duck OR Chicken OR Turkey OR Quail OR Crow OR Migratory wild birds OR Resident Wild bird OR Human) AND Bangladesh (Table 1). The search field option was selected as “All fields.” A restriction was placed on the language of publication, “English.” Search terms and keywords were adjusted according to minor differences in the syntax rules of five electronic databases. All articles were listed in a spreadsheet, and duplication was removed by using the reference management software EndNote X8 (Clarivate Analytics, Philadelphia, PA, USA). The reference lists of extracted articles were also searched manually in triplicate for additional potential articles and to ensure that selected database searches did not miss any reports. We excluded some articles including only abstracts, studies conducted outside of Bangladesh, species out of poultry and humans, and articles in foreign languages. We also excluded the reports on AIV surveillance activities by GoB and NGOs. After thorough searching, we've identified 94 relevant articles for our comprehensive review and analysis.

## 3. Poultry Production Systems in Bangladesh

The Food and Agriculture Organization (FAO) broadly defines four types of poultry production systems, including backyard production, small-scale commercial production, large-scale commercial production, and industrial and integrated production [44], and all these systems are available in Bangladesh. Commercial poultry farming can be categorized based on farm size as large-scale breeder farming, medium-sized farming (broiler, layer, and duck farming), and small-sized farming (layer, broiler, quail, pigeon, turkey, and guinea fowl farming) [45]. Industrial poultry has gained popularity by adopting modern and high-yielding breeds, strains, and variants in the last two decades, due to increasing community demand for poultry products [46]. Commercial poultry meat and egg farms are currently the most important poultry product source. At present, there are 65,000–70,000 commercial layer and broiler farms, including 16 grant parents stocks and 206 breeder farms have been listed in Bangladesh [45, 47]. However, this sector coexists with a large number (over 81% of total farms) of small-scale poultry farms (poultry population ≤ 2,000) [42]. The contribution of the poultry industry includes the production of 385.7 million live poultry which contribute 35.25% of total meat products and 23,376.3 million eggs in 2022–2023 livestock production in Bangladesh [46, 48]. Before the outbreak of HPAIV A/H5N1 in 2007, this sector gained around a 15%–20% annual production growth rate [45]. LBMs are the main hub of live poultry trading in Bangladesh and they account for more than 90% of the total live poultry [49].

## 4. Avian Influenza in Different Poultry Production Systems

**Figure 1:**
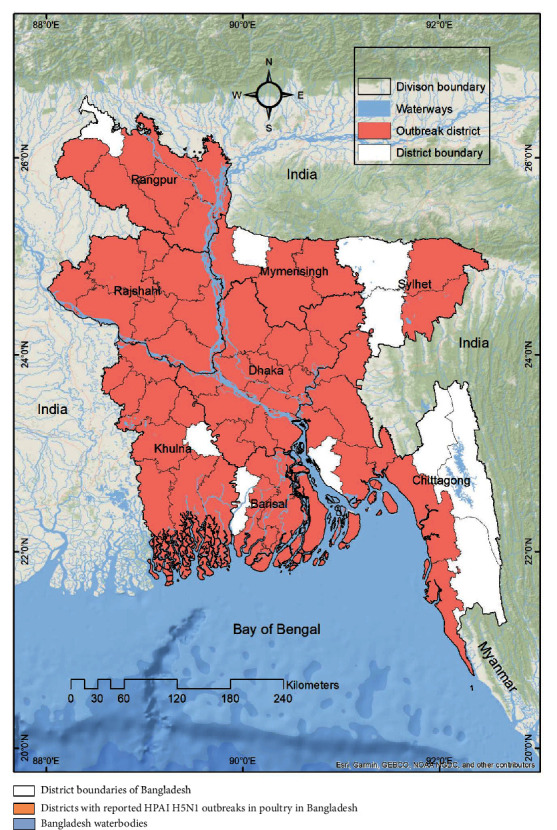
The distribution of HPAIV H5N1 outbreak in poultry reported districts in Bangladesh. The map shows the district boundaries of Bangladesh, where indicates the districts with reported HPAI H5N1 outbreaks in poultry and country's waterbodies.

From the beginning of the H5N1 outbreak in 2007 in Bangladesh [50], 585 outbreaks have been reported in 54 of the 64 districts (Figure 1) throughout the country till now [51, 52]. In Bangladesh, most of the HPAIV outbreaks were reported in small-scale commercial poultry farms [53]. In Bangladesh, 83 outbreaks of HPAIV A/H5N1 were reported during 2007–2012 in the commercial poultry system, which declined to 2% from 2013 to 2019 [54]. During the first wave of HPAIV A/H5N1 in 2007 (February 2007–July 2007), 3.1% of the commercial farms were affected, which was 22.7% during the second wave in 2008 (January 2008–April 2008) in the central (Dhaka division) and northwestern part (Rajshahi division) of Bangladesh [33]. A total of 31% of subdistricts (148 out of 481) were officially confirmed with the HPAIV A/H5N1 outbreak in Bangladesh during 2007–2008, which was 6.03% of subdistricts during the first wave and 23.90% during the second wave [33].

**Figure 2:**
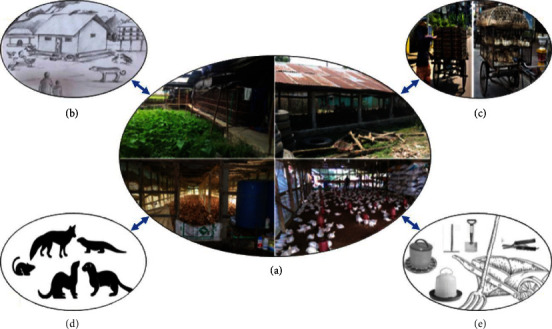
Transmission dynamics of avian influenza virus in the different poultry production systems of Bangladesh. (a) Shows four different poultry farm settings, which also shows poor biosecurity management in the farms. The virus can move from commercial farms to backyard poultry farms or vice versa by stray dogs, cats, resident birds, and human workers (b). The virus can spread through the live poultry and egg transportation system (c), resident wild animals, such as foxes, mink, ferrets, rodents, and monitor lizards (Guishap) (d) and farm tools (e) [55].

Different poultry-rearing systems and species have been shown to play different roles in the transmission and maintenance of AIVs in the country (Figure 2) [56, 57]. From the beginning of the HPAIV A/H5N1 outbreak epidemic in Bangladesh, commercial and backyard poultry farming systems were affected, particularly backyard chickens, which are highly susceptible to HPAIV A/H5N1 infection as these birds have close contact with domestic ducks and wild birds [58].

In backyard poultry, the HPAI H5N1 was reported first in 2007 [59]. The AIV viral prevalence in backyard poultry has been recorded by different studies, ranging from 0.94% to 25.2% [60, 61]. The viral prevalence was 3.2%–42.31% among the household chickens [61, 62]. Different levels of viral prevalence in domestic ducks in different parts of Bangladesh were 2.4%–24.9% in different districts of Bangladesh [63–66]. By AIV subtype in Bangladesh, the viral prevalence of AIV infection in the cloacal sample of domestic ducks was 2.1%–9% for A/H9 and 1.4%–89% for A/H5 [62, 67, 68]. The seroprevalence of AIV infection for A/H5 and A/H9 was 71.4% and 28.5% in household chickens, and 76.3% and 36.8% in domestic ducks, respectively, in Tanguar and Hakaluki hoars [69]. Several biosecurity factors such as offering slaughter remnants of purchased chickens in the backyard, having water bodies within 1 km, contact with migratory birds and other domestic poultry species (duck, pigeons), wild animals (rodents), keeping the chicken and ducks at the same place in the night and presence of commercial farms close to the backyard are responsible to boost the AIV transmission in the backyard poultry [58].

Unlike backyard production systems, a lower level of AIV prevalence has been recorded in commercial production systems. The viral prevalence of AIV was reported at 7.73%–14.2% in commercial poultry farms where HPAIV H5 and LPAIV H9 were detected at 31.1% and 6.67%, respectively [2, 70]. In commercial layer farms, the prevalence of AIV was reported 64.04% with 49.12% HPAIV H5N1 and 15.79% LPAIV H9N2 farm-level infection [20] The AIV prevalence was 1.8% in commercial broiler chickens and 1.6%–1.9% in commercial layer chickens, respectively, in Chattogram and Cox's Bazar districts during 2017, where the AIV A/H9N2 detection rate was 0.5%–0.6% [54].

Because of the larger number of encounters with intermediaries such as traders, suppliers, and transporters and the absence of physical obstacles to infection, small-scale commercial farms may be at a higher risk than backyard poultry and large industrial poultry farms [42, 71]. The decline of the HPAIV A/H5N1 outbreak in poultry in Bangladesh may be due to low reporting of the H5N1 outbreaks in Bangladesh, where farmers are disposing of their own sick or dead flocks with suspected AIV without informing the local and official veterinarians [20]. Recent confirmed H5N1 AIV isolates were not observed as pathotyped, causing mortality between 1.05% and 5.5%, confirmed as a newly reassorted H5N1 clade 2.3.2.1a [20].

## 5. Influence of Live Bird Marketing System in Avian Influenza Transmission

**Figure 3:**
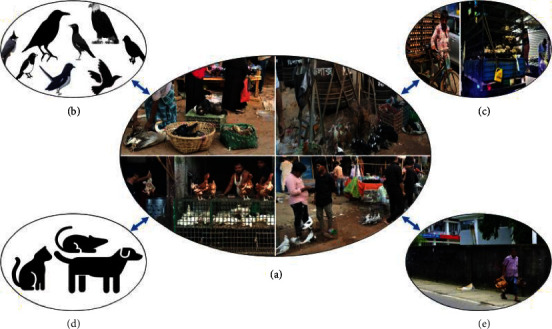
Transmission dynamics of avian influenza virus in the live bird marketing system of Bangladesh. The central figure (a) shows the open live bird marketing systems in Bangladesh, either by small shops or street marketing. The virus can spread through (b) the resident birds such as crow, sparrow, magpie, starling, bulbul, and eagle; (c) egg and live bird transportation systems; (d) stray animals, such as dogs, cats, and commensal rodents; and (e) mobile poultry vendor in the urban area [55].

In Bangladesh, the poultry supply chain is comprised of a complex network of intermediaries, including (1) the LBMs of commercial and backyard poultry in both urban local or roadside markets and established markets in metropolitan areas, (2) the movements of wild and resident birds in the LBMs and farms, (3) the large-scaled transportation of eggs and live poultry from farms to markets, (4) the presence of dogs, cats, and rodents in the LBMs for searching for poultry's offal, and (5), the home selling of live poultry to consumers through vendors (Figure 3). LBM is the hub of poultry trading, where both wholesale and retail marketing are available, and multispecies birds from commercial and backyard poultry farms, and occasionally resident wild and migratory birds are brought and housed together with higher concentrations for sale [39, 72]. Such marketing systems are available across the country, with a higher number in the main cities like Dhaka and Chattogram [49, 73]. Market facilities are, therefore, a suitable niche for the persistence and perpetuation of AIV transmission from sick or carrier birds to humans and other poultry [10, 51, 68, 74].

Detection of AIV RNA in environmental samples of LBMs indicates AIV infection in carcasses, offal, and excreta of birds, increasing the risk of transmission to healthy birds, vendors, other poultry workers/contractors, and consumers [75]. Moreover, the presence of local avian predators such as crows in LBMs might also pose a biosecurity risk at LBMs [76, 77]. Since 2008, several subtypes of AIVs, including HPAIV A/H5N1 and LPAIVA/H9N2, have been detected in LBMs in Bangladesh [8, 68, 78]. Different studies have reported LBMs level AIV prevalence as 4.4%–82.5% in Bangladesh where LBMs infected with HPAIV A/H5 and HPAIV A/H9 subtype were 1.9%–23.8% and 1.6%–63.2%, respectively [2, 22, 35, 73, 79, 80]. The prevalence of positive poultry stalls at LBMs was reported at 29%–50% in different districts of Bangladesh, where the H5 and H9 subtypes of AIV were reported at 2.8%–10.5% and 3.1%–14%, respectively [68, 73, 80, 81]. In Bangladesh, most of the stalls in the LBMs are kept open daily, with no proper electricity and drainage systems. Besides, multiple poultry species and strains of poultry are kept together [80, 81], which resulted in a higher prevalence of AIV in LBMs and creates a suitable environment for transmitting and amplifying AIV more frequently and allows spreading of the virus over a large geographic area [22, 73]. Most of the stalls keep the unsold birds overnight at the shop in the same place selling them [80, 81].

## 6. Avian Influenza in Wetland Ecosystems

**Figure 4:**
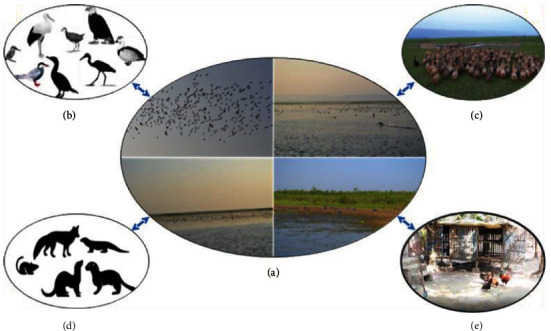
Transmission dynamics of avian influenza virus in the wetland ecosystem of Bangladesh. The virus is imported to Bangladesh wetlands by the migratory bird (a). Then the virus can spread throughout Bangladesh by the resident wild birds, such as the cormorant, heron, water hen, egal, kingfisher, and wild duck (b); range ducks (c), resident wild animals (d), and domestic ducks in the backyard poultry farming system (e) [55].

Bangladesh is located in the Bengal Basin (the world's largest delta), reported to have 7–8 million hectares of resourceful wetlands, which are created by major rivers traversing the country (Padma, Brahmaputra, and Meghna) [82] and the seacoast with widespread mangrove belts. These wetlands are the most productive ecosystem in Bangladesh, cultivating paddy, fish, and poultry with other aquatic flora and fauna [83], which are suitable and essential sites for wild migratory birds, specially the *Anseriformes* birds of the Arctic and Palearctic regions of Russia, during the winter season. Moreover, the wetlands of Bangladesh lie in two of the major migratory bird flyways: the eastern edge of the Central Asian and the western edge of the East Asian-Australian routes [82, 84, 85]. During winter, those migratory birds come in close contact with resident, nomadic, and household birds and share the same water bodies of the open wetlands of Bangladesh. Migratory birds usually enter Bangladesh in November and December, which led to the hypothesis that migratory birds might have played a role in the initial introduction of the virus to domestic and commercial poultry in Bangladesh [30, 33, 86]. The interaction of migratory waterfowl and free-range poultry, such as domestic ducks and chickens in these agroecological habitats, increases the risk of AIV crosstransmission in the country [87]. Domestic duck remains asymptomatic and sheds influenza viruses, which play an important role in transmitting the virus to other poultry species (such as chicken, turkey, and so forth) when they share a common husbandry system [88–90]. Besides this, nomadic ducks in the wetlands of Bangladesh are considered subjunctive species between wild or migratory birds and domestic poultry, which might transmit and spread the infection [91–93]. Indeed, the wetlands of Bangladesh are now well recognized as the hub of AIV introduction and spread in Bangladesh (Figure 4). The seroprevalence of AIV in wetlands was reported to be 39.6%–90.2% in different wetlands throughout Bangladesh over time [63, 94, 95]. Besides, the RNA prevalence of AIV was reported 0.5%–39.8% in different wetlands throughout the country [63, 66, 90, 91, 96]. Besides domestic birds, AIV has been identified in wild birds all over the world [97, 98].

Wild birds are often considered the main pathway for the spread of AIV, although there is no distinct evidence in support of this claim [69, 77]. AIVs are naturally found in wild waterfowl and migratory birds, and they are responsible for spreading the virus to domestic and wild birds [98]. Every year during the winter season, a large influx of migratory birds, estimated to be around 50,000 individuals from 60 different species, swarm to Bangladesh [99], especially in the wetlands reach areas, where they mix with domestic and resident wild birds [69]. Migratory birds in those wetlands are believed to have played a crucial role in the initial introduction of HPAIV A/H5N1 into Bangladesh, leading to the initiation of outbreaks in domestic poultry [3, 33]. Transmission of AIV between LBMs and wild birds has been reported due to the low biosecurity in the LBMs [39]. Besides wild migratory birds, house crows are also affected by the H5N1 virus [76, 100]. House crows are found scattered in every place close to human residence which can transmit the virus from one place to another place. Besides, the seroprevalence of AIV has been detected in resident birds in Bangladesh which is also a possible source of transmission of AIV infection as the roosting site of those resident birds is close to the wetlands in Bangladesh [69]. Till now, 10 AIV HA serotypes have been detected in wild migratory birds and five serotypes in wild resident birds [69]. The seroprevalence of AIV has been calculated as 9.7%, including 0% for H5 and 6.6% for H9 in captive wild birds of safari parks and zoos in Bangladesh from November 2013 to February 2014 [98], 65.9% for H5 and 56% for H9 in wild migratory birds, and 66.7% for H5 and 30.6% for H9 in resident wild birds in Bangladesh during the winter season of 2012–2014 [69]. Besides this, RNA prevalence was detected as 1.95% during the winter seasons of 2009–2011 and 2011–2012 [38], and 17.5% during 2005–2006 [101] in wild migratory birds in Bangladesh. According to the findings of those studies, wild migratory birds are a major source of AIV introduction and spreading to domestic birds and LBMs in Bangladesh.

## 7. Molecular Epidemiology of Avian Influenza in Bangladesh

Multiple clades of the HPAIV A/H5N1 virus were identified in poultry across South Asia from January 2003 to December 2018, where the most common identified clade was 2.2 [102]. Since the first detection of HPAIV A/H5N1, three different clades of HPAI H5N1 have been detected in Bangladesh [103, 104], including 2.2.2, 2.3.4.2, and 2.3.2.1a [2, 86, 93]. The circulating AIV belonged to clade 2.2, causing multiple outbreaks in poultry and humans that were closely identical to the isolates of the neighboring countries, including India and Bhutan [11]. After that, new clades, 2.3.2.1 and 2.3.4, were introduced in Bangladesh in 2011, affecting the different poultry production systems [105]. Between 2007 and 2010, HPAIV A/H5N1 circulated in Bangladesh belonging to clade 2.2, which became endemic in the country during this period [106]. At the beginning of 2011, a new invasion of HPAIV A/H5N1 clades 2.3.2.1 and 2.3.4.2 was detected in chickens, ducks, crows, quails, turkeys, and migratory birds in Bangladesh, resulting in multiple deaths in poultry [105, 107–109]. Ducks were not noticed with any clinical signs before 2011[108]. However, with the introduction of HPAIV A/H5N1 clades 2.3.2.1, infected ducks showed nervous signs and 10%–47% mortality [67, 108]. However, new clade 2.3.2.1a of HPAIV is responsible for present outbreaks of AIV in both poultry and humans in Bangladesh [12, 104]. The HA genes of AIV H5N1 clade 2.3.2.1a shifted into nine genetic subgroups from R1 to R9 where only the R9 subgroup is circulating in Bangladesh and subcategories R1–R8 were not detected after 2016 [66, 77]. The clade 2.3.2.1 was also reported with diarrhea, drowsiness, and 13% mortality in turkeys [109], and sudden convulsions and 100% mortality in quails [108]. Multiple circulating clades and subclades of HPAIV and LPAIV in different poultry species in Bangladesh indicate the continuous evolution through genetic drift and shift [110]. The HI titers of recent H5N1 isolates were identified as lower than the homologous titer, indicating the occurrence of antigenic drift among H5N1 isolates [103]. Antigenic shift among AIV due to the extensive host and reservoir range and jumping across the species barrier, considering the source of antigenically distinct viruses [111, 112]. It may be due to the increasing interaction of different poultry species such as sharing of the same feeding habitat of domestic ducks and migratory birds in wetlands [66, 96], and mixing of domestic ducks and other poultry species in LBMs where domestic ducks play an important role in the maintenance and development of new reassorting viruses [22, 79]. On the different aspect, introducing vaccination against LPAIV and HPAIV in commercial poultry increases the vaccination-built herd immunity, which may impose a selection pressure and enforce viral antigenic drift resulting in the escape of vaccination-induced immunity and continuing the spreading of drift variants into the poultry species [110, 112], reported in other countries such as China, Egypt, Indonesia, Hong Kong, and Vietnam [113, 114]. The variants of AIV carry the amino acids replacement, mainly at the HA epitopes along with NP genes as it is the major part of identifying species specificity [115]. H5N1 isolates of Bangladesh had the cleavage site of the basic amino acid at the position of 321–333 of the HA gene [103], which is considered a marker for highly pathogenic in chickens [116]. The PQRERRRKR/GLF CS motif was identified in the majority of the detected isolates of the 2.3.2.1 clade, including deletion at position 329, which is common in clade 2.3.2.1 H5N1 viruses from Bangladesh and neighboring countries: India and Nepal, as well as wild birds from China, Mongolia, and Tyva in 2009 and 2010. Apart from that, a few isolates exhibited an amino acid change at position 323 of the HA gene [103]. All the H5N1 viruses had a 20-amino acid deletion at the positions of 49–68 in the NA region, which is required to adapt virus transmission from wild aquatic birds to domestic chickens [117]. The PB2 E627K mutation, which is important for mammalian host adaptation, was present in the H5N1 isolates from clade 2.2.2, but not found in any of the viruses from clade 2.3.2.1 isolated in Bangladesh as well as PB2 amino acid residue 702R in clade 2.3.2.1 was present in most of the H5N1 viruses from Bangladesh and India isolated in 2011 or later [103]. Most of the H5N1 isolates from clades 2.2.2 and 2.3.2.1 had a five amino acid deletion at the positions 80–85 in the NS protein, which is also present in other HPAIVs isolated after 2001 [103]. Still now seven HA genetic subgroups (B1–B7) and six genotypes (G1, G1.1, G1.2, G2, G2.1, and G2.2) have been noticed in Bangladesh, but only the B7 subgroup and G2, G2.1, and G2.2 genotypes were detected after 2016. After the introduction of vaccination in 2012, the genetic diversity of HPAIV H5N1 increased, and the G1 genotype was replaced by the G2 genotype [118]. The LPAIV H9N2 belongs to the G1 lineage, which is endemic in chickens in Bangladesh [6, 8], and shares the internal genes with HP viruses of subtypes H7N3 and H5N1[5]. Reassortments of the AIV have been reported between H9N2 and H5N1, H7N3 and H7N9 subtypes [7]. Most of the identified H9N2 viruses in Bangladesh exhibited the Q234L substitution and also contained amino acid residues N166D, H191, and E399K located within the receptor binding site of the HA protein [5], which favors the transmission of H9N2 to mammalian hosts by respiratory droplets [119]. Till now two different lineages (G1 and Kr) of H9N2 subtypes have been introduced in Bangladesh, resulting in five variations in the HA cleavage site, four host-specific mutations in mammalian hosts at the receptor binding sites, and 10 different patterns of glycosylation sites [5].

## 8. Human Cases of Avian Influenza in Bangladesh

It is commonplace for rural communities in Bangladesh to raise poultry in backyard systems that include the management, feeding, slaughtering, and care of sick poultry without hygienic and biosecurity precautions [120]. Such practices place rural communities at risk of zoonotic AIVs [12]. Since the first detection of AIV in humans in 2008 [121, 122], 11 human cases have been reported in Bangladesh, where eight were caused by HPAIV A/H5N1 and three were LPAIV A/H9N2 [110]. Of the eight human cases of HPAIV A/H5N1, three caused the mild illness that was detected through population-based surveillance in 2008 [123]. Human cases of AIV in Bangladesh were reported to persons who had direct contact with LBMs. The risk of animal-to-human HPAIV A/H5N1 virus transmission is unclear among poultry workers as the clinical signs include mild illness and asymptomatic in humans [124]. Two HPAIV A/H5N1 cases were reported in young children (a boy and a girl of 13 and 31 months of age, respectively) who had direct contact with poultry, where HPAIV A/H5N1 was recorded in crows and LBMs of that locality [123]. Another three HPAIV A/H5N1 human cases were reported in LBM's poultry workers that remained in close contact with sick birds, practiced inadequate hygiene management such as sanitation and disinfections, used no personal protective equipment (PPE), and had low awareness about zoonotic transmission of AIV[125]. The first human LPAIV A/H9N2 subtype infection in Bangladesh was identified in 2011, belonging to the G1 lineage, and characterized by fever, headache, runny nose, cough, and sneezing [126]. The only fatal case of AIV was a 23-month-old boy who was reported in 2013, had severe pneumonia, meningitis, and disseminated intravascular coagulation, and died within a week of admission. The boy had a history of close contact with sick backyard chickens and the infection was confirmed with 2.3.2.1 clades of HPAIV A/H5N1[127]. Laboratory testing reports suggested that the patient was infected with HPAIV A/H5N1 [122]. Identification of AIV A/H5 and A/H9 in LBMs in Bangladesh might lead to genetic reassortment and evolution of new AIV strains in poultry and human populations [9]. The beginning of the AIV infection in humans belonged to clade 2.2 of HPAIV in Bangladesh during 2007–2008 [121]. Human infection with clade 2.2.2 in 2011, 2.3.2.1 in the fatal case of 2012, and during 2015–2019 a new clade 2.3.2.1a virus was detected in Bangladesh [9, 128].

## 9. Intervention Strategies to Combat Avian Influenza in Bangladesh

### 9.1. Biosecurity in the Poultry Value Chain

During the early outbreaks of HPAIV A/H5N1 in poultry, commercial poultry farms were characterized by low biosecurity practices, including an inappropriate choice of location for a farm establishment, no restriction of wild bird and animal access, no/improper fencing, and no use of footbaths [129, 130]. However, commercial poultry farm owners are frequently exchanging equipment and materials (egg trays, feeders, and waterers) [131]. Improvement of poultry market chain biosecurity has been demonstrated to be the best disease control and prevention strategy to limit the transmission of AIVs [41]. At the farm level, such practices include restricting visitors and vehicles to the farms, cleaning and disinfecting the poultry sheds and equipment, and using PPE while handling poultry [132]. In addition, restriction of contact between wild bird populations and poultry is an effective way to prevent viral transmission from the natural reservoir into poultry facilities [34, 50]. The frequent movement of free-ranging birds in the wetlands of Bangladesh exposes them to more contact with wild and migratory birds in the winter season, leading to a higher infection rate of AIVs [93]. Some specific areas should be fixed as sanctuaries for migratory birds, and measures need to be taken to not intermingle domestic poultry with migratory birds in wetlands, especially during the winter season [101].

However, recommended biosecurity measures are poorly maintained in endemic countries like Bangladesh [17, 41, 42, 133]. Farmers of different poultry production systems have different decision-making processes for interventions for AIV infection. Commercial poultry farmers are strongly influenced by the implementation of farm biosecurity rather than backyard poultry farmers [132]. Currently, large-scale commercial farms are equipped with solid biosecurity measures and maintain the recommendation. However, small-scale commercial farms still lack proper biosecurity practices. Therefore, they face acute financial impacts due to sudden AIV outbreaks [12]. Social culture does not influence people to follow hygienic measures, such as washing hands during the handling of backyard poultry [132]. In this regard, raising awareness among backyard poultry raisers should be the focus [22]. Information on biosecurity measures and practices for different poultry production systems should be shared through mass and social media, meetings, and campaigns to prevent an outbreak and reduce the effect of AIV on both poultry and humans. At the same time, the biosecurity guidelines of the GoB for poultry farming must be enforced and monitored by authorities at all levels of the poultry supply chain [110].

LBMs act as important hubs in the poultry market chain network and as a potential source of viral transmission among different poultry and poultry traders [68]. The hygiene practices and poultry management at LBMs play an important role in AIV infection. Low biosecurity, a lack of infrastructure, and awareness of zoonotic transmission, prevention, and risk assessment with AIV often follow in LBMs in Bangladesh [12]. Waste products of LBMs, including offal's, feathers, and blood of slaughtered poultry, are disposed of in a dustbin, drains, and water bodies like ponds, rivers, or roadsides, and vendors of LBMs keep their leftover birds in their shops for the next day's sale, which contaminate the surrounding environment and spread the risk of AIV infection [73]. LBMs setup with rest days and disinfection interventions were reported as having the effect of discontinuing virus circulation in LBMs [134] though the GoB imposed an order in 2012 to practice it within Dhaka [79].

### 9.2. Avian Influenza Vaccination Program

In the absence of adequate AIV biosecurity in the poultry value chain, AIV vaccination is an effective intervention to reduce the animal health and welfare impacts of AIV infection, although therapeutic options for treating influenza virus infections are minimal [135]. Apart from its individual-level animal health and welfare advantages, AIV vaccination triggers the reduction of virus shedding and, as a result, decreases the viral load in the environment at the poultry–human interface [21, 136]. As in many endemic countries, Bangladesh resorts to vaccination against HPAIV, primarily in the commercial poultry sector [17]. Initially, three vaccines were introduced on an experimental basis in these two districts [110]. Currently, two licensed vaccines against H5N1, RE-6 (an inactivated vaccine) and rHVT-H5 (recombinant herpesvirus of Turkey with an H5 insert) are applied for commercial use on larger commercial broiler and layer farms in Bangladesh [16]. A recent H5N1 surveillance study reported that the level of anti-H5 seropositivity was similarly low in both vaccinated and unvaccinated chickens, suggesting the low effectiveness of the vaccine against H5N1 [60]. However, vaccination failure can be observed due to inappropriate administration due to incorrect dose, route, and accurate age. Some other factors, such as frequent changes in antigenic drift due to impaired immunity in vaccinated poultry, immune escape mutation, and inappropriate cool chain, limit the effectiveness of the vaccination program [16–19]. Vaccination programs against LPAI H9N2 have also been approved recently in Bangladesh by the DLS, and the vaccine (inactivated CEVAC FLU H9 K) is available in the market. Yet the entire backyard sector and a majority of the small-scale farms remain unvaccinated against AIVs in Bangladesh [110].

### 9.3. Avian Influenza Surveillance Program in Poultry and Humans

Disease monitoring and surveillance are critical for early detection of HPAIV A/H5N1 and quick response to mitigate the outbreak in poultry and the environment [17, 133]. To identify current AIV strains circulating in Bangladesh's poultry sector, different surveillance programs have been initiated since 2007 with the support of the International Centre for Diarrhoeal Disease Research, Bangladesh (icddr, b), where funding and technical support is provided by the US Centers for Disease Control and Prevention (CDC) in collaboration with DLS, primarily targeting LBMs and domestic poultry within Bangladesh[12]. Since the HPAI H5N1 outbreak in commercial and backyard poultry farms, passive surveillance has been strengthened, and initiated active surveillance to detect HPAIV A/H5N1 rapidly in 2008 by DLS as part of the influenza preparedness and response plan [12]. This initiative continued until 2013 when DLS got and reviewed the internet-based short message service about the disease, morbidity, and mortality of poultry from poultry farm owners [12]. In 2016, a new environmental surveillance activity, “sink surveillance” was initiated by the animal and health services of GoB in collaboration with FAO to detect AIV in pooled environmental samples of LBMs in Dhaka and Chattogram, which later was extended in other cities of Bangladesh [12]. Furthermore, the DLS with the support of FAO in 2019 adopted the Upazila-to-Community program with an operational goal of a prospective strategy for control of HPAIV A/H5N1 in Bangladesh [12]. The goal was to protect poultry in farms and villages to decrease the prevalence of HPAIV A/H5N1 by implementing environmental sampling within LBMs in Dhaka and Chattogram metropolitan areas, trial village surveillance program in villages being surveyed twice a week, and deployment of rapid detection tests if HPAIVs are suspected, and including participatory disease surveillance techniques [12].

Besides the above-mentioned surveillance activities, GoB approved stamping out in HPAIV A/H5N1 infected poultry flock, which was partly been successful in the early outbreak [17, 133], but this control measure was stopped due to the discontinuation of compensation by GoB [25, 132]. FAO-GoB active surveillance program was also supported by a compensation policy to the HHPAIV A/H5N1 affected farmers at the beginning of the AI outbreak during 2007–2008. This compensation policy was stopped later in 2012, which might be a cause of the current underreporting of the AIV outbreak in Bangladesh. Although nowadays GoB adopted isolation of HPAIV-infected flocks having lower mortality is practiced in Bangladesh, with additional stamping out followed in case of higher mortality [110], the majority (>51%) of backyard poultry raisers in Bangladesh were reluctant to follow the recommendation of the GoB as they did not recognize the zoonotic transmission of HPAIV to human, and as disease threat to their poultry like other endemic poultry diseases such as Newcastle disease in Bangladesh [43].

In humans, hospital-based influenza surveillance for AIV infection detection was initiated in 2007 by icddr,b in collaboration with the GoB National Institute named Institute of Epidemiology Disease Control And Research (IEDCR) and international partner US-CDC [137]. Another human surveillance program named “National Influenza Surveillance” was started in 2010 by the IEDCR to identify the circulation of AIV strains in humans in Bangladesh [137]. Surveillance for detection of AIV infection in LBMs poultry was started in 2012 by icddr, b in partnership with IEDCR and DLS, which performed as a “One Health” platform both for poultry and market workers [12]. To strengthen awareness, GoB launched a national mass media campaign, including radio, television, newspapers, and public conferences, to disseminate ten suggested recommendations for preventing AIV infection in humans [138].

### 9.4. One Health Movement

The GoB developed and applied the First National Avian Influenza and Human Pandemic Influenza Preparedness and Response Plan (NAIRP) with the support of the World Health Organization (WHO) and FAO, covering the sessions of 2006–2008 [139] and the second plan in a period of 2009–2011 [140]. This plan was adopted by a multisectoral approach led by the Ministry of Health and Family Welfare (MoH & FW), the Ministry of Fisheries and Livestock (MoFL), and the Ministry of Environment and Forestry (MoEF) of the GoB with other stakeholders, including international organizations, national and international NGOs, trade association, and poultry farmers [12]. The United Nations International Children's Emergency Fund (UNICEF) helped GoB to develop a risk communication strategy, and the United States Agency for International Development managed the financial support in different aspects to control HPAIV [12]. The Department of Mass Communication of the Ministry of Information and Broadcasting (MoIB), in collaboration with DLS, carried out public awareness and information campaigns about AIVs through mass media [12]. In May 2016, a Chatham House roundtable was convened in Dhaka with the government, international multilateral organizations, NGOs, and trade associations in Bangladesh to discuss the future policy to control and prevent AIV and other poultry zoonotic diseases in Bangladesh [12]. Future policy of AI prevention should be under the One Health umbrella by engaging multiple sectors, including poultry production stockholders, GoB bodies (MoH & FW, MoFL, MoIB, and MoEF), international multilateral organizations (FAO, WHO, World Bank, and UNICEF), World Organization for Animal Health (OIE), CDC, and NGOs (such as Bangladesh Centre for Communication Programs, Bangladesh Rural Advancement Committee, EcoHealth Alliance, and icddr,b) should be recommended [25]. Practical steps should be taken by partner organizations working together according to the NAIRP to prevent and control AIVs in Bangladesh. As part of a government platform, One Health can serve as a centralized hub for activities in AIV combat including joint surveillance and outbreak investigations in both poultry and human, and advocacy of AIV control and prevention with the collaboration of MoH & FW, MoFL, MoIB, and MoEF. The collaborative works may be monitored from a common platform like One Health Secretariat and maintained a dashboard to share the data. The capacity to detect, investigate, and respond to AIV outbreaks, and to coordinate these efforts within a One Health framework, could be improved by adopting a One Health approach. In assisting the control and prevention of AIV, a One Health platform can facilitate transdisciplinary research into the origins of disease and issues at the interface among humans, poultry, and ecosystems. Besides, it can foster networking and partnerships among government and other key stakeholders in preventing and controlling AIV at the local, national, regional, and global levels. It can facilitate and develop the knowledge, attitudes, and skills among the poultry raisers and workers about the control and prevention of AIV and help to assess their situations and to act protect their health, livelihoods, and environment against AIV. Additionally, One Health workforce can establish the technical and logistical capacity to enable government, partners, and key stakeholders to prevent, detect, and respond to AIV at poultry, human, and environmental interfaces.

## 10. Conclusions

The poultry industry supplies the principal source of protein in Bangladesh's diet. In the past 15 years, the circulation of both zoonotic HPAIV A/H5N1 and LPAIV A/H9N2 throughout the country has resulted in several poultry outbreaks, causing severe economic losses to Bangladesh's poultry industry. Emerging new strains, subtypes, and clades in different domestic poultry species, including wild birds and migratory birds, continue to pose a risk to poultry and humans in Bangladesh. The risk of zoonotic transmission to humans is still a public health concern, as human mortality has been reported in Bangladesh with HPAIV A/H5N1 infection. People involved directly with the poultry sector in Bangladesh, including farmers, transporters, sellers, and workers in both backyard and LBMs, remain at risk of exposure due to a lack of adequate compliance with biosecurity protocols. Even though HPAIV A/H5N1 and LPAIV A/H9N2 are found in different poultry sectors, the two strains occur in divergent clusters within Bangladesh, and their prevalence varies even between different locations and species. Currently, HPAIV A/H5N1 infections are rarely reported in Bangladesh, which may be associated with poor case detection. Disease monitoring, early detection of AIVs, culling, vaccination, and biosecurity are important to reduce the viral load and control AIV outbreaks. Targeted risk-based active surveillance is the key initial step to preparing for and responding to future AIV outbreaks. Monitoring the reassortments of viral subtypes is important to identify dominant strains within the poultry sector and assess the risk of human infection. Besides this, public awareness of food-chain biosecurity interventions should be improved through educational programs, advertisements in mass media, and discussion sessions with officials. Finally, an effective and well-coordinated One Health policy for AIV in Bangladesh will not only reduce public health risk as well as economic losses of future outbreaks.

## Figures and Tables

**Table 1:** Algorithm for electronic database search to find published reports on the narrative reports of avian influenza viruses in Bangladesh.

Search term	Boolean keywords
Descriptiveterm	Avian Influenza OR Bird Flu OR HPAI OR LPAI OR H5 OR H9 OR H5N1 OR H9N2

Populationterm	LBM OR Live bird market OR Backyard poultry OR Household poultry OR Commercial poultry OR Broiler OR Layer OR Wild bird OR Migratory wild birds OR Duck OR Chicken OR Turkey OR Quail OR Crow OR Resident Wild bird OR Human

Area	Bangladesh

## Data Availability

No data is available for this study.
